# Supplementary material for: Habitual Choline Intakes across the Childbearing Years: A Review
Source: Nutrients. 2021 Dec 8;13(12):4390. doi: 10.3390/nu13124390 (PMC8709092; doi:10.3390/nu13124390)

Supplemental Table S1. Search key terminologies.

**Population – Women of Childbearing Age**
"childbearing" [tiab] OR "pregnan*" [tiab] OR "periconcept*" [tiab] OR "lactat*" [tiab] OR maternal [tiab] OR "mother*" [tiab] OR "women" [tiab] OR "females" [tiab] OR "adults" [tiab] OR "pregnant women"[Mesh] OR "Breast Feeding"[Mesh] OR "lactation" [Mesh]
**Habitual Choline Intakes from habitual food and supplementation sources**
"choline nutri*" [tiab] OR "choline intake" [tiab] OR "choline diet*" [tiab] OR "choline food*" [tiab]
**Excluded Publications** Mouse [tiab] OR mice [tiab] OR rat [tiab] OR rats [tiab] OR murine [tiab] OR bovine [tiab]
**Final Search #1 AND #2 NOT #3**

Supplementary Figure S1: Non-pregnant women.

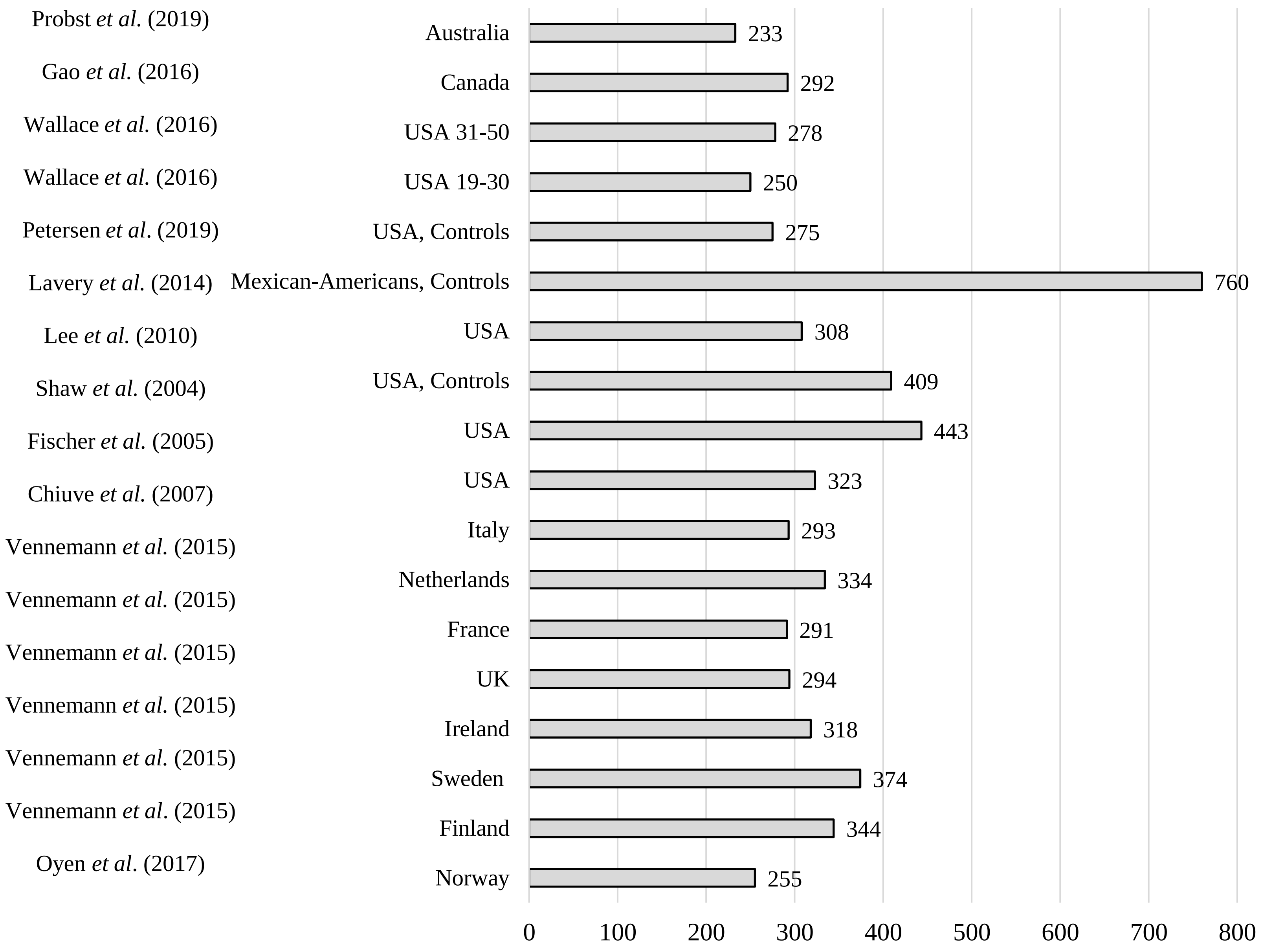

Supplementary Figure S2; Pregnancy.

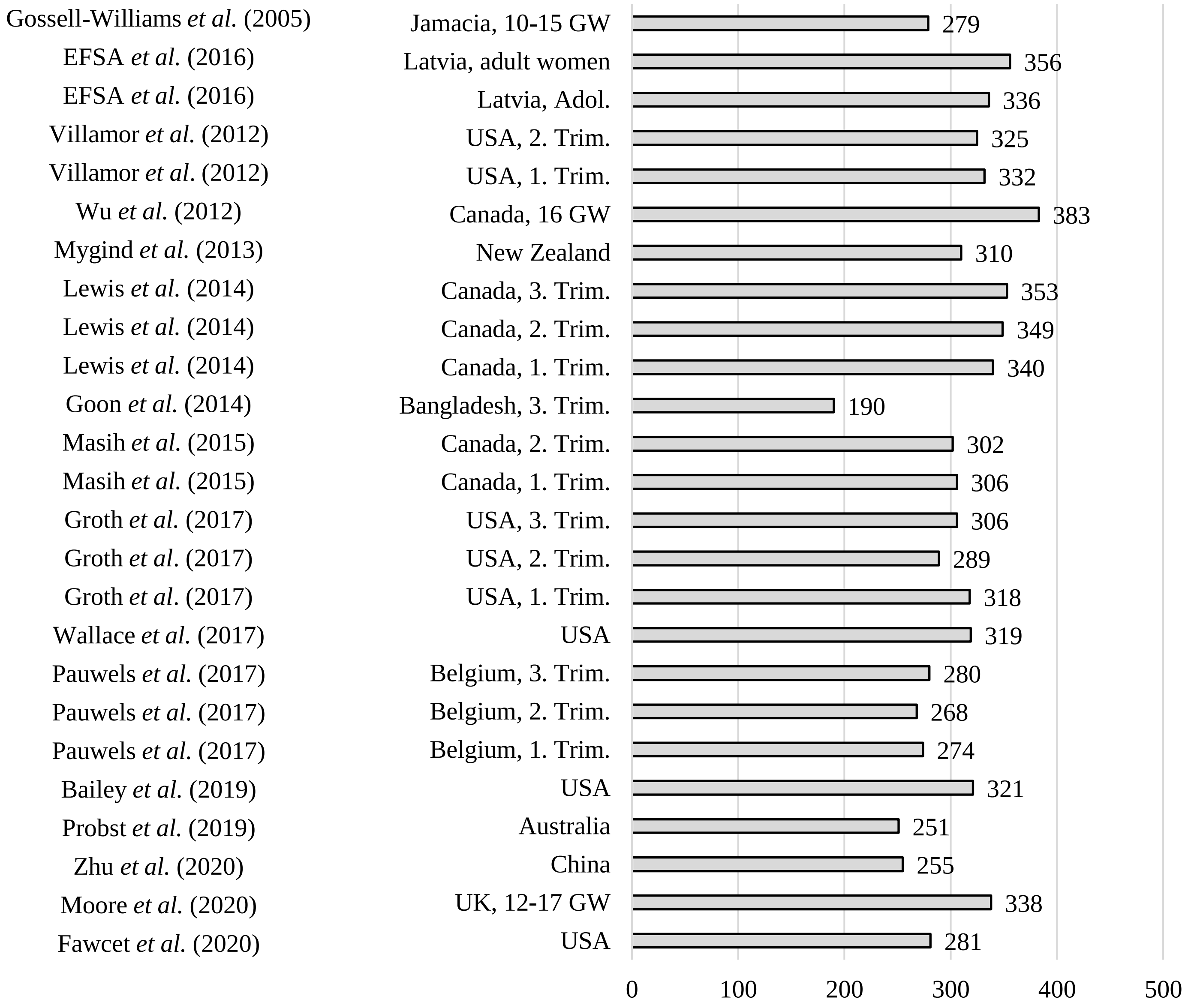

Supplementary Figure S3; Lactation

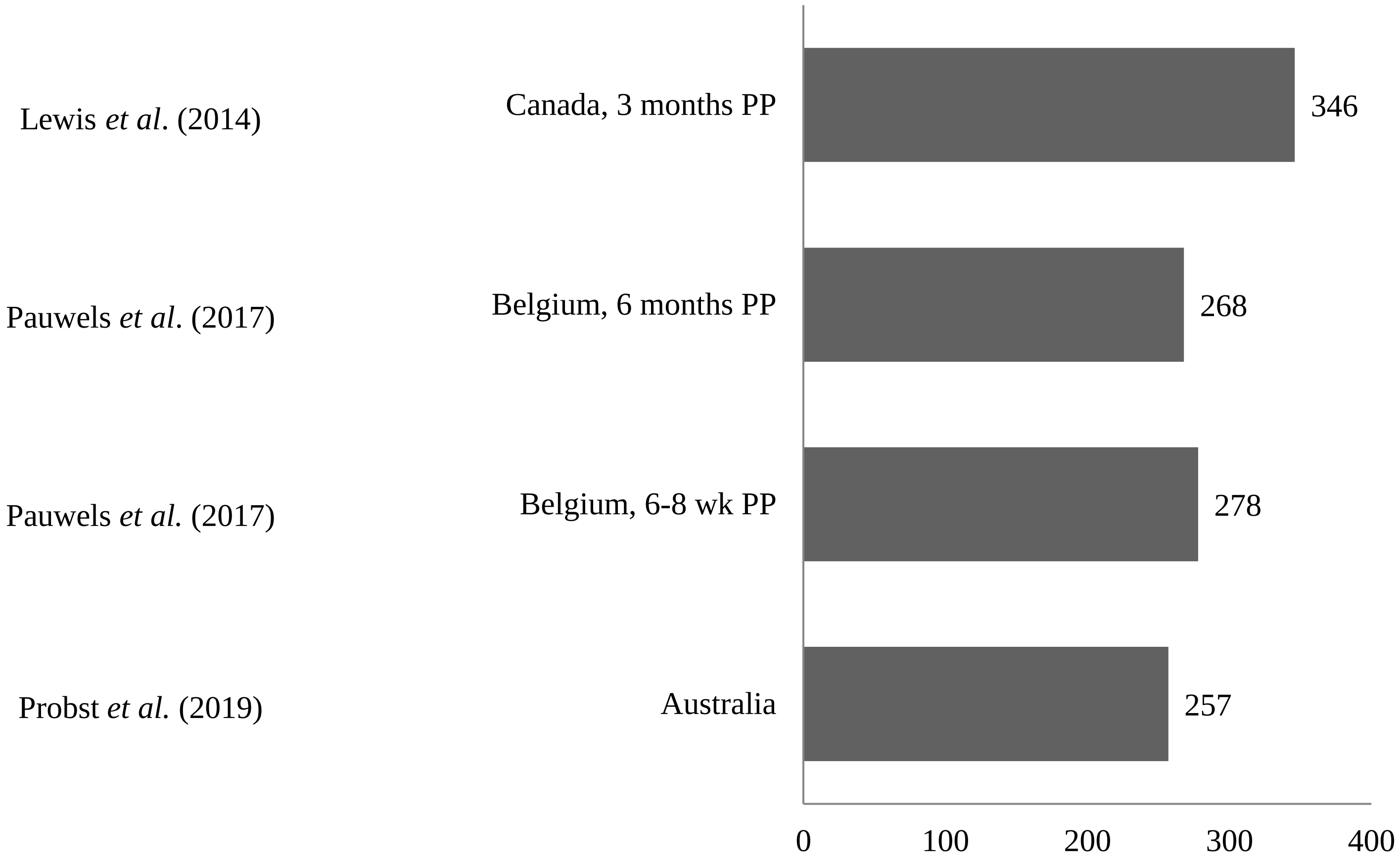

Supplement: Supplementary file 1 [file nutrients-13-04390-s001.zip › nutrients-1461714-supplementary.pdf]
